# Impact of atopy on risk of glioma: a Mendelian randomisation study

**DOI:** 10.1186/s12916-018-1027-5

**Published:** 2018-03-15

**Authors:** Linden Disney-Hogg, Alex J. Cornish, Amit Sud, Philip J. Law, Ben Kinnersley, Daniel I. Jacobs, Quinn T. Ostrom, Karim Labreche, Jeanette E. Eckel-Passow, Georgina N. Armstrong, Elizabeth B. Claus, Dora Il’yasova, Joellen Schildkraut, Jill S. Barnholtz-Sloan, Sara H. Olson, Jonine L. Bernstein, Rose K. Lai, Minouk J. Schoemaker, Matthias Simon, Per Hoffmann, Markus M. Nöthen, Karl-Heinz Jöckel, Stephen Chanock, Preetha Rajaraman, Christoffer Johansen, Robert B. Jenkins, Beatrice S. Melin, Margaret R. Wrensch, Marc Sanson, Melissa L. Bondy, Richard S. Houlston

**Affiliations:** 1 Division of Genetics and Epidemiology, The Institute of Cancer Research, 15 Cotswold Road, London, SM2 5NG UK; 2 Department of Medicine, Section of Epidemiology and Population Sciences, Dan L. Duncan Comprehensive Cancer Center, Baylor College of Medicine, Houston, TX USA; 3 Case Comprehensive Cancer Center, School of Medicine, Case Western Reserve University, Cleveland, OH USA; 4 Division of Biomedical Statistics and Informatics, Mayo Clinic College of Medicine, Rochester, MN USA; 5 School of Public Health, Yale University, New Haven, CT USA; 6 Department of Neurosurgery, Brigham and Women’s Hospital, Boston, MA USA; 7 Department of Epidemiology and Biostatistics, School of Public Health, Georgia State University, Atlanta, GA USA; 8 Duke Cancer Institute, Duke University Medical Center, Durham, NC USA; 9 Cancer Control and Prevention Program, Department of Community and Family Medicine, Duke University Medical Center, Durham, NC USA; 10 Department of Epidemiology and Biostatistics, Memorial Sloan Kettering Cancer Center, New York, NY USA; 11 Departments of Neurology and Preventive Medicine, Keck School of Medicine, University of Southern California, Los Angeles, CA USA; 12 Department of Neurosurgery, University of Bonn Medical Center, Sigmund-Freud Str. 25, 53105 Bonn, Germany; 13 Human Genomics Research Group, Department of Biomedicine, University of Basel, Basel, Switzerland; 14 Department of Genomics, Life & Brain Center, University of Bonn, Bonn, Germany; 15 Institute of Human Genetics, University of Bonn School of Medicine & University Hospital Bonn, Bonn, Germany; 16 Institute for Medical Informatics, Biometry and Epidemiology, University Hospital Essen, University of Duisburg-Essen, Essen, Germany; 17 Division of Cancer Epidemiology and Genetics, National Cancer Institute, Bethesda, USA; 18 Institute of Cancer Epidemiology, Danish Cancer Society, Copenhagen, Denmark; 19 Rigshospitalet, University of Copenhagen, Copenhagen, Denmark; 20 Department of Laboratory Medicine and Pathology, Mayo Clinic Comprehensive Cancer Center, Mayo Clinic, Rochester, MN USA; 21 Department of Radiation Sciences, Umeå University, Umeå, Sweden; 22 Department of Neurological Surgery, School of Medicine, University of California, San Francisco, San Francisco, CA USA; 23 Institute of Human Genetics, University of California, San Francisco, CA USA; 24 Sorbonne Universités UPMC Univ Paris 06, INSERM CNRS, U1127, UMR 7225, ICM, F-75013 Paris, France; 25 AP-HP, Groupe Hospitalier Pitié-Salpêtrière, Service de neurologie 2-Mazarin, Paris, France; 26 Division of Molecular Pathology, The Institute of Cancer Research, London, UK

**Keywords:** Mendelian randomisation, Allergy, Cancer, Glioma, Risk

## Abstract

**Background:**

An inverse relationship between allergies with glioma risk has been reported in several but not all epidemiological observational studies. We performed an analysis of genetic variants associated with atopy to assess the relationship with glioma risk using Mendelian randomisation (MR), an approach unaffected by biases from temporal variability and reverse causation that might have affected earlier investigations.

**Methods:**

Two-sample MR was undertaken using genome-wide association study data. We used single nucleotide polymorphisms (SNPs) associated with atopic dermatitis, asthma and hay fever, IgE levels, and self-reported allergy as instrumental variables. We calculated MR estimates for the odds ratio (OR) for each risk factor with glioma using SNP-glioma estimates from 12,488 cases and 18,169 controls, using inverse-variance weighting (IVW), maximum likelihood estimation (MLE), weighted median estimate (WME) and mode-based estimate (MBE) methods. Violation of MR assumptions due to directional pleiotropy were sought using MR-Egger regression and HEIDI-outlier analysis.

**Results:**

Under IVW, MLE, WME and MBE methods, associations between glioma risk with asthma and hay fever, self-reported allergy and IgE levels were non-significant. An inverse relationship between atopic dermatitis and glioma risk was found by IVW (OR 0.96, 95% confidence interval (CI) 0.93–1.00, *P* = 0.041) and MLE (OR 0.96, 95% CI 0.94–0.99, *P* = 0.003), but not by WME (OR 0.96, 95% CI 0.91–1.01, *P* = 0.114) or MBE (OR 0.97, 95% CI 0.92–1.02, *P* = 0.194).

**Conclusions:**

Our investigation does not provide strong evidence for relationship between atopy and the risk of developing glioma, but findings do not preclude a small effect in relation to atopic dermatitis. Our analysis also serves to illustrate the value of using several MR methods to derive robust conclusions.

**Electronic supplementary material:**

The online version of this article (10.1186/s12916-018-1027-5) contains supplementary material, which is available to authorized users.

## Background

Although glioma accounts for approximately 80% of malignant primary brain tumours [1], to date, few aetiological risk factors are well established for the disease [2]. Over the past three decades the search for an immune-mediated risk factor that might influence risk has led to studies of a possible relationship between multiple allergic conditions and autoimmune disorders with glioma [3].

Several case-control studies have shown that self-reported allergic conditions may protect against glioma [4]. For example, in the International Adult Brain Tumour Study, based on 1178 glioma patients, an odds ratio (OR) of 0.59 was found for any self-reported allergy [5]. Other case-control studies have reported similar ORs, however, most have been reliant on substantial numbers of proxy informants (up to 44%) [4, 6] and have potential bias as a consequence of how controls were ascertained, thereby casting doubt on findings. In contrast to case-control studies, evidence for an association between glioma and allergy from cohort-based analyses has been less forthcoming [7], although such studies have been poorly powered to demonstrate a relationship.

Assaying IgE potentially reduces bias stemming from self-reporting despite levels not necessarily corresponding to specific allergies or equating to a single allergic response. Nevertheless, measurement of IgE has been explored by a number of researchers seeking to identify risk factors for glioma [8–10]. In a case-control study of 228 cases and 289 controls performed in 2004 [8], self-reported allergies and IgE levels were both inversely associated with glioma, but concordance between the two outcomes was poor. In a larger study of 535 cases and 532 controls [11], both self-reported allergies and IgE levels were inversely related to glioma risk; however, IgE levels in patients were affected by temozolomide treatment. A case-control study nested within the European Prospective Investigation into Cancer and Nutrition cohort based on prospectively collected serum IgE levels reported a non-significant OR of 0.73 [9]. A similar nested case-control study performed in the USA based on 181 cases reported a non-significant OR of 0.72 for high serum IgE [10].

Several mechanisms have been proposed to explain a possible association between atopic disease and glioma [12]. The findings could reflect a true causal effect of the heightened immune function reported for atopy on tumour development. Alternatively, the associations observed might be non-causal, arising as a consequence of methodological biases inherent in the study design. Imprecisely defined exposures, such as allergic disease, are likely to have affected the validity of the findings of both case-control and cohort studies. The heterogeneous description of allergy in studies and different levels of detail in self-reporting on individual allergies complicate the interpretation of results. Additional biases include possible selection bias in controls, recall bias from self-reported allergy assessment and reverse causation or confounding from unmeasured effects. Finally, the high frequency of exposure ascertainment by proxy for cases is also likely to have systematically biased findings.

Mendelian randomisation (MR) analysis can be used to minimise potential biases in conventional observational studies and to determine the causal association of an exposure with an outcome such as disease risk [13]. The causal association can also be manifested by common genetic and biological pathways that determine two sequentially developed phenotypes such as an atopic trait and glioma risk. Atopy has a strong heritable basis [14, 15] and, thus far, genome-wide association studies (GWAS) have identified over 50 loci associated with different atopy-related traits [16]. The alleles associated with atopy should be randomly assigned to offspring from parents during mitosis, a process analogous to the random assignment of subjects to an exposure of interest in randomised clinical trials. Thus, genetic scores summarising the effects of single nucleotide polymorphisms (SNPs) associated with atopy-related traits can serve as instrumental variables (IVs) in a MR analysis of atopy and glioma risk.

To examine the nature of the association between atopy and glioma, we implemented two-sample MR [17] to estimate associations between atopy-associated SNPs and glioma risk using summary data from the recent GWAS meta-analysis performed by the Glioma International Case-Control Consortium study [18].

## Methods

Two-sample MR was undertaken using GWAS data. Ethical approval was not sought for this specific project because all data came from the summary statistics of published GWAS, and no individual-level data were used.

### Glioma genotyping data

Glioma genotyping data were derived from the most recent meta-analysis of GWAS in glioma, which related > 10 million genetic variants (after imputation) to glioma, in 12,488 glioma patients and 18,169 controls from eight independent GWAS datasets of individuals of European descent [18] (Additional file 1: Table S1). Comprehensive details of the genotyping and quality control of the seven GWAS have been previously reported [18].

### Genetic variant instruments for atopic traits

SNPs associated with each of the atopy-related traits investigated, namely atopic dermatitis (eczema), asthma and hay fever, IgE level, and self-reported allergy, by the NHGRI-EBI GWAS Catalog [19–26] at genome-wide significance (*i.e. P* ≤ 5.0 × 10^− 8^) in individuals with European ancestry were used as IVs. To avoid co-linearity between SNPs for each trait, we excluded SNPs that were correlated (*i.e.* r^2^ value of ≥ 0.001) within each trait, and only considered the SNPs with the strongest effect on the trait for use as IVs (Additional file 2: Table S2). For each SNP, we recovered the chromosome position, risk allele, association estimates (*per*-allele log-OR) and standard errors (Table 1). The allele that was associated with increased risk of the exposure was considered the effect allele. For IgE level, the allele associated with an increase in serum IgE was considered the effect allele. Allele frequencies for these SNPs were compared between the atopy-related trait and glioma datasets to ensure that the effect estimates were recorded with respect to the same allele. Gliomas are heterogeneous and different tumour subtypes, defined in part by malignancy grade (e.g. pilocytic astrocytoma World Health Organization (WHO) grade I, diffuse ‘low-grade’ glioma WHO grade II, anaplastic glioma WHO grade III and glioblastoma (GBM) WHO grade IV) can be distinguished [27]. For the sake of brevity we considered gliomas as being either GBM or non-GBM.

**Table 1:** Variant and effect allele with frequencies and magnitude of effect on each atopy-related trait and strength of association with glioma

Region	SNP	Position (bp)^a^	Alleles^b^	MAF	Hay fever and asthma	Glioma
					OR (95% CI)	OR (95% CI)
2q12.1	rs10197862	102,966,549	G/A	G = 0.161	1.24 (1.16–1.32)	0.98 (0.93−1.03)
4p14	rs4833095	38,799,710	C/T	T = 0.425	1.20 (1.14–1.26)	1.03 (0.99−1.08)
5q22.1	rs1837253	110,401,872	T/C	T = 0.382	1.17 (1.11–1.23)	0.96 (0.93−1.00)
8q21.13	rs7009110	81,291,879	C/T	C = 0.467	1.14 (1.09–1.19)	0.98 (0.94−1.01)
9p24.1	rs72699186	6,175,855	A/T	T = 0.110	1.26 (1.17–1.36)	0.97 (0.93−1.02)
11q13.5	rs2155219	76,299,194	G/T	G = 0.468	1.17 (1.13–1.21)	1.01 (0.97−1.05)
15q22.33	rs17294280	67,468,285	A/G	G = 0.120	1.18 (1.12–1.25)	0.98 (0.94−1.03)
16p13.13	rs62026376	11,228,712	T/C	T = 0.144	1.17 (1.11–1.23)	0.97 (0.93−1.01)
17q21.1	rs7212938	38,122,680	T/G	G = 0.473	1.16 (1.11–1.22)	1.00 (0.97−1.04)
Region	SNP	Position^a^	Alleles^b^	MAF	Atopic dermatitis	Glioma
					OR (95% CI)	OR (95% CI)
1q21.3	rs11205006	152,440,176	T/A	A = 0.265	1.62 (1.48–1.77)	0.96 (0.91−1.02)
1q21.3	rs2228145	154,426,970	A/C	C = 0.293	1.15 (1.10–1.20)	0.99 (0.96−1.03)
2p25.1	rs10199605	8,495,097	A/G	A = 0.244	1.04 (1.03–1.06)	1.01 (0.97−1.05)
2p13.3	rs112111458	71,100,105	G/A	G = 0.224	1.08 (1.05–1.10)	0.98 (0.92−1.03)
2q24.3	rs6720763	167,992,286	T/C	C = 0.320	1.29 (1.18–1.41)	1.02 (0.97−1.06)
5p13.2	rs10214237	35,883,734	C/T	C = 0.176	1.06 (1.05–1.08)	0.98 (0.94−1.02)
5q31.1	rs1295686	131,995,843	C/T	T = 0.422	1.35 (1.22–1.49)	0.99 (0.95−1.03)
6p21.32	rs12153855	32,074,804	T/C	C = 0.125	1.58 (1.40–1.78)	0.97 (0.92−1.03)
8q21.13	rs6473227	81,285,892	A/C	A = 0.473	1.06 (1.05–1.08)	0.98 (0.94−1.02)
9p21.3	rs10738626	22,373,457	C/T	C = 0.397	1.23 (1.15–1.32)	0.96 (0.93−1.00)
10p15.1	rs6602364	6,038,853	G/C	G = 0.492	1.05 (1.03–1.07)	1.03 (0.99−1.07)
11q13.1	rs10791824	65,559,266	A/G	G = 0.490	1.15 (1.12–1.19)	0.99 (0.95−1.02)
11q24.3	rs7127307	128,187,383	C/T	C = 0.488	1.09 (1.07–1.11)	0.99 (0.95−1.03)
11q13.5	rs7130588	76,270,683	G/A	G = 0.216	1.29 (1.20–1.38)	1.02 (0.98−1.06)
14q13.2	rs2143950	35,572,357	C/T	T = 0.215	1.08 (1.06–1.10)	1.01 (0.97−1.06)
16p13.13	rs2041733	11,229,589	C/T	T = 0.496	1.09 (1.06–1.11)	0.97 (0.94−1.01)
19p13.2	rs2164983	8,789,381	C/A	A = 0.169	1.16 (1.10–1.22)	0.95 (0.90−1.00)
20q13.33	rs909341	62,328,742	T/C	T = 0.262	1.32 (1.21–1.44)	1.32 (1.26−1.37)
Region	SNP	Position^a^	Alleles^b^	MAF	IgE level^c^	Glioma
					OR (95% CI)	OR (95% CI)
1q23.2	rs2251746	159,272,060	C/T	C = 0.015	1.09 (1.08–1.11)	0.98 (0.95−1.02)
5q31.1	rs20541	131,995,964	A/G	A = 0.270	1.08 (1.06–1.10)	1.01 (0.97−1.06)
6p22.1	rs2571391	29,923,838	C/A	C = 0.303	1.06 (1.05–1.08)	0.97 (0.94−1.01)
6p21.32	rs2858331	32,681,277	A/G	G = 0.490	1.04 (1.03–1.06)	1.02 (0.98−1.06)
12q13.3	rs1059513	57,489,709	C/T	C = 0.070	1.13 (1.09–1.17)	0.97 (0.92−1.03)
Region	SNP	Position^a^	Alleles^b^	MAF	Self–reported allergy	Glioma
					OR (95% CI)	OR (95% CI)
2q12.1	rs10189699	102,879,464	A/C	A = 0.143	1.16 (1.12–1.20)	0.99 (0.94−1.04)
2q33.1	rs10497813	198,914,072	T/G	T = 0.401	1.08 (1.05–1.11)	0.99 (0.96−1.03)
3q28	rs9860547	188,128,979	G/A	A = 0.272	1.08 (1.05–1.11)	1.02 (0.98−1.06)
4p14	rs2101521	38,811,551	A/G	A = 0.475	1.15 (1.12–1.18)	1.02 (0.98−1.07)
4q27	rs17388568	123,329,369	G/A	A = 0.141	1.08 (1.05–1.11)	1.01 (0.97−1.05)
5p13.1	rs7720838	40,486,896	G/T	T = 0.362	1.08 (1.06–1.11)	1.02 (0.99−1.06)
5q22.1	rs1438673	110,467,499	T/C	C = 0.296	1.12 (1.09–1.15)	0.97 (0.94−1.01)
6p21.33	rs9266772	31,352,113	T/C	C = 0.175	1.11 (1.08–1.14)	1.03 (0.98−1.08)
9p24.1	rs7032572	6,172,380	A/G	G = 0.114	1.12 (1.08–1.16)	0.97 (0.93−1.02)
10p14	rs962993	9,053,132	T/C	T = 0.106	1.07 (1.05–1.10)	1.02 (0.98−1.06)
11q13.5	rs2155219	76,999,194	G/T	G = 0.468	1.11 (1.09–1.14)	1.01 (0.97−1.05)
15q22.33	rs17228058	67,450,305	A/G	G = 0.100	1.08 (1.05–1.11)	1.00 (0.96−1.04)
17q21.1	rs9303280	38,074,031	T/C	T = 0.346	1.07 (1.05–1.09)	0.98 (0.94−1.02)
20q13.2	rs6021270	50,141,264	C/T	T = 0.346	1.16 (1.10–1.22)	1.02 (0.94−1.10)

^a^NCBI build 37

^**b**^Reference allele/effect allele

^c^Per standard deviation

*MAF* minor allele frequency, *OR* odds ratio, *SNP* single nucleotide polymorphism

### Two-sample MR method

The association between each atopy-related trait and glioma was examined using MR on summary statistics using the inverse-variance weighting (IVW) method and maximum likelihood estimation (MLE) as *per* Burgess *et al.* [28]. The IVW ratio estimate $\left(\widehat{\upbeta}\right)$ of all SNPs associated with each atopy-related trait on glioma risk was calculated as follows:

$$ \widehat{\upbeta}=\frac{\sum_k{X}_k{Y}_k{\sigma_Y}_k^{-2}}{\sum_k{X}_k^2{\sigma_Y}_k^{-2}} $$

Where *X*_*k*_ corresponds to the association of SNP *k* (as log of the OR per risk allele) with the atopy-related trait *Y*_*k*_ is the association between SNP *k* and glioma risk (as log OR) with standard error ${\sigma}_{Y_k}$. The estimate for $\left(\widehat{\upbeta}\right)$ represents the causal increase in the log odds of glioma for each trait. The standard error of the combined ratio estimate is given by:

$$ se\left(\widehat{\upbeta}\right)=\sqrt{\frac{1}{\sum_k{X}_k^2{\sigma_Y}_k^{-2}}} $$

For the MLE, a bivariate normal distribution for the genetic associations was assumed, and the R function *optim* was used to estimate β. $se\left(\widehat{\upbeta}\right)$ was calculated using observed information. The correlation between the errors of *Y*_*k*_ and *X*_*k*_ was taken to be 0 as they were derived from independent studies.

A central tenet in MR is the absence of pleiotropy (*i.e.* a gene influencing multiple traits) between the SNPs influencing the exposure and outcome disease risk [13]. This would be revealed as deviation from a linear relationship between SNPs and their effect size for atopy and glioma risk. To examine for violation of the standard IV assumptions in our analysis we first performed MR-Egger regression, as well as HEIDI-outlier analysis, as per Zhu *et al.* [29], imposing the advocated threshold of *P* ≤ 0.01. Additionally, we derived weighted median estimates (WME) [30] and mode-based estimates (MBE) [31] to establish the robustness of findings.

Atopic dermatitis, asthma and hay fever, and self-reported allergy as well as all of the disease outcomes (all glioma, GBM and non-GBM glioma) are binary. The causal effect estimates therefore represent the odds for outcome disease risk per unit increase in the log OR of the exposure disease [32]. These ORs were converted to represent the OR for the outcome disease per doubling in odds of the exposure disease to aid interpretation [32].

For each statistical test we considered a global significance level of *P* < 0.05 as being satisfactory to derive conclusions. To assess the robustness of our conclusions, we initially imposed a conservative Bonferroni-corrected significance threshold of 0.0125 (*i.e.* 0.05/4 atopy-related traits). We considered a *P* value ≥ 0.05 as non-significant (*i.e.* no association), a *P* < 0.05 as evidence for a potential causal association, and a *P* < 0.0125 as significant evidence for an association. All statistical analyses were undertaken using R software (Version 3.1.2). The meta and gsmr packages were used to generate forest plots and perform HEIDI-outlier analysis [29].

The power of a MR investigation depends greatly on the proportion of variance in the risk factor that is explained by the IV. We estimated study power *a priori* using the methodology of Burgess *et al.* [33], making use of published estimates of the heritability of trait associated IV SNPs [34–36], as well as estimates found by direct calculation (Additional file 3: Table S3), and the reported effect of each trait on glioma risk reported in a meta-analysis of epidemiological studies [18]. Additional file 4: Table S4 shows the range of ORs for which we had less than 80% power to detect for each of the four atopy-related traits.

### Simulation model

Through simulation we evaluated the suitability of using each employed MR method in a two-sample setting with binary-exposure and binary-outcome data. Let *i* index genetic variants, *N* be the total number of genetic variants, and *j* index individuals. Genetic variants *g*_*ij*_ were generated independently by sampling from a Binomial(2,*p*_*j*_) distribution with probability *p*_*j*_ drawn from a Uniform(0.1,0.9) distribution, to mimic bi-allelic SNPs in Hardy–Weinberg equilibrium. Let *w*_*j*_ correspond to the per-allele OR for the exposure disease, sampled from ORs reported for genome-wide significant SNPs reported in the GWAS Catalog [37], and *v* be the OR for the outcome disease per doubling in odds of the exposure disease. For each individual, exposure disease odds *x*_*j*_, outcome disease odds *y*_*j*_, exposure disease status *a*_*j*_, and outcome disease status *b*_*j*_ were determined as follows:

$$ {x}_j={x}_0\prod \limits_{i=1}^N{w_i}^{g_{ij}} $$

$$ {y}_j={y}_0\times {2}^{{\mathsf{\log}}_2{x}_j\times {\mathsf{\log}}_2v} $$

$$ {a}_j\sim \mathsf{Binomial}\left(1,\frac{x_j}{1+{x}_j}\right) $$

$$ {b}_j\sim \mathsf{Binomial}\left(1,\frac{y_j}{1+{y}_j}\right) $$

Data for 1,000,000 individuals were simulated and partitioned at random to reflect the two-sample setting. Cases and controls for the exposure and outcome GWAS were sampled from each half of the dataset using the exposure and outcome disease statuses of each individual, and association statistics computed under an additive logistic regression model. To ensure the simulated data closely resembled the atopy-related trait and glioma data, the simulation analysis was repeated for each binary atopy-related trait using the same number of genetic variants as IVs and the same numbers of case and control individuals as used to estimate the atopy-related trait and glioma association statistics (Additional file 5: Table S5). Parameters *x*_*0*_ = 0.0005 and *y*_*0*_ = 0.01 were chosen to ensure the prevalence of the simulated exposure and outcome diseases were similar to that of the atopy-related traits and glioma, respectively (Additional file 5: Table S5). To determine the suitability of each MR method we considered two scenarios: (1) no causal relationship between exposure and outcome (*v* = 1.00) and (2) a causal relationship between exposure and outcome (*v* = 1.33). We performed 100 simulations for each scenario for each binary atopy-related trait.

## Results

The atopic dermatitis risk SNP rs909341, which is highly correlated with the chromosome 20q13.33 glioma risk SNP rs2297440 (D’ = 0.89, r^2^ = 0.77), was strongly associated with risk of glioma (*P* = 2.10 × 10^−34^). Testing for pleiotropy using HEIDI-outlier analysis formally identified rs909341 as violating the assumption of the instrument on the outcome. Henceforth, we confined our analysis of the relationship between atopic dermatitis and glioma to a dataset excluding this SNP.

**Fig. 1:**
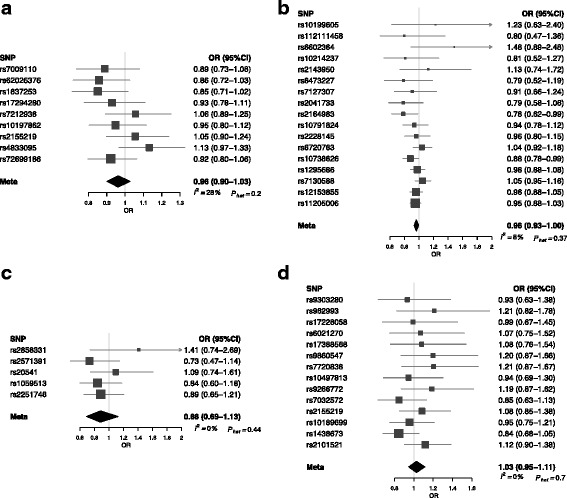
Forest plot of Wald odds ratios (ORs) and 95% confidence intervals generated from single nucleotide polymorphisms (SNPs) associated with atopy-related traits. ORs for individual SNPs are listed according to magnitude of effect in the instrumental variable analysis and are presented with pooled effects using the inverse-variance weighting method. Squares represent the point estimate, and the bars are the 95% confidence intervals. **a** Asthma and hay fever, **b** atopic dermatitis, **c** IgE level, **d** self-reported allergy

Figure 1 shows forest plots of ORs for glioma generated from the SNPs. There was minimal evidence of heterogeneity between variants for asthma and hay fever, atopic dermatitis, IgE levels and self-reported allergy (respective *I*^2^ and *P*_het_ values being 28% and 0.192, 8% and 0.377, 0% and 0.444, and 0% and 0.707). Including rs909341 in the analysis for atopic dermatitis, the *I*^2^ value was 90% and *P*_het_ < 10^− 4^ (Additional file 6: Figure S1), providing further evidence that inclusion of this SNP would invalidate the MR analysis.

The results of the IVW, MLE, WME, MBE and MR-Egger methods are summarised in Table 2. Using the IVW method to pool results from individual SNPs, no associations (*i.e. P ≥* 0.05) were identified between genetically conferred risk of raised IgE level (OR 0.88, 95% CI 0.69–1.13, *P* = 0.319), asthma and hay fever (OR 0.96, 95% CI 0.90–1.03, *P* = 0.248), or self-reported allergy (OR 1.03, 95% CI 0.95–1.11, *P* = 0.534) with risk of all glioma. There was some support for an inverse relationship between atopic dermatitis and glioma risk (OR 0.96, 95% CI 0.93–1.00, *P* = 0.041), albeit not significant after adjustment for multiple testing.

**Table 2:** Inverse-variance weighting, maximum likelihood estimation, weighted median estimate, mode-based estimate and Mendelian randomisation-Egger test results for combined atopy-related instrumental variables

Trait	IVW		MLE		WME		MBE		MR-Egger slope		MR-Egger intercept	
	OR (95% CI)	*P*	OR (95% CI)	*P*	OR (95% CI)	*P*	OR (95% CI)	*P*	OR (95% CI)	*P*	Estimate (95% CI)	*P*
Asthma and hay fever	0.96 (0.90–1.03)	0.248	0.96 (0.93–1.00)	0.066	0.93 (0.86–1.01)	0.087	0.91 (0.80–1.04)	0.191	0.99 (0.72–1.36)	0.951	−0.007 (−0.030 to 0.016)	0.542
Atopic dermatitis	0.96 (0.93–1.00)	0.041	0.96 (0.94–0.99)	0.003	0.96 (0.91–1.01)	0.114	0.97 (0.92–1.02)	0.194	0.97 (0.92–1.03)	0.375	0.004 (−0.014 to 0.006)	0.396
IgE level	0.88 (0.69–1.13)	0.319	0.88 (0.74–1.05)	0.157	0.83 (0.61–1.12)	0.218	0.82 (0.57–1.19)	0.355	0.63 (0.32–1.25)	0.184	0.027 (0.001 to 0.053)	0.042
Self-reported allergy	1.03 (0.95–1.11)	0.534	1.02 (0.97–1.08)	0.429	1.08 (0.97–1.20)	0.184	1.12 (0.92–1.36)	0.275	0.92 (0.69–1.22)	0.540	0.017 (0.003 to 0.031)	0.018

*CI* confidence interval, *IVW* inverse-variance weighting, *MBE* mode-based estimate, *MLE* maximum likelihood estimation, *MR* Mendelian randomisation, *OR* odds ratio, *WME* weighted median estimate

**Fig. 2:**
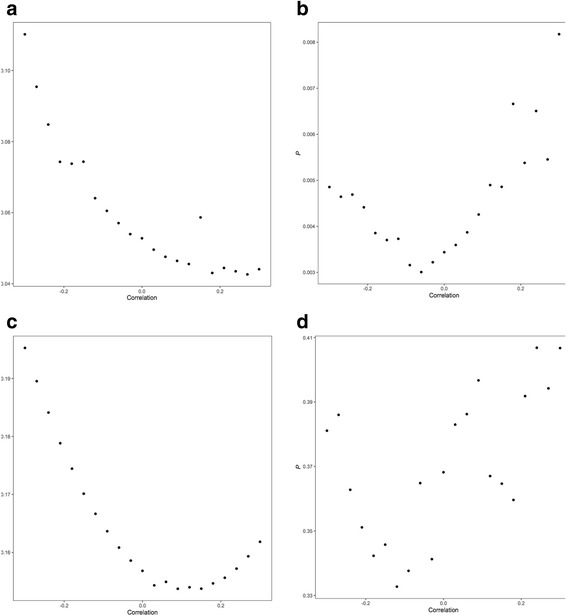
Plot of *P* value of maximum likelihood estimation associations with glioma against correlation between errors in *X*_*k*_ and *Y*_*k*_. **a** Asthma and hay fever, **b** atopic dermatitis, **c** IgE level, **d** self-reported allergy

Using MLE, no associations were identified between asthma and hay fever (OR 0.96, 95% CI 0.93–1.00, *P* = 0.066), IgE levels (OR 0.88, 95% CI 0.74–1.05, *P* = 0.157) or self-reported allergy (OR 1.02, 95% CI 0.97–1.08, *P* = 0.429) with risk of all glioma. For atopic dermatitis, an OR of 0.96 (95% CI 0.94–0.99, *P* = 0.003) was shown, which remained significant after adjusting for multiple testing. Figure 2 shows relaxation of the assumption that the correlation between the errors in *X*_*k*_ and *Y*_*k*_ is zero for each of the atopy-related traits demonstrating the consistency of findings. Specifically, for a correlation in the range −0.15 to 0.15, the association between atopic dermatitis and glioma risk remained significant.

In contrast to findings from IVW and MLE, no significant support was provided by either the WME or MBE for an association between any of the atopy-related traits and glioma risk, including atopic dermatitis (WME: OR 0.96, 95% CI 0.91–1.01, *P* = 0.114; MBE: OR 0.97, 95% CI 0.92–1.02, *P* = 0.194; Table 2).

**Fig. 3:**
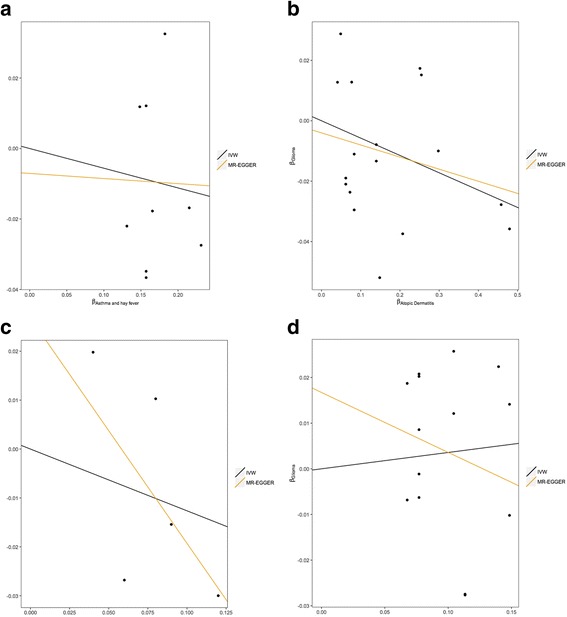
Scatter plots of genetic associations with glioma against genetic associations with the exposure. **a** Asthma and hay fever, **b** atopic dermatitis, **c** IgE level, **d** self-reported allergy

The respective effect estimated from MR-Egger regression (Fig. 3) were 0.97 for atopic dermatitis (95% CI 0.92–1.03; *P* = 0.375), 0.63 for IgE levels (95% CI 0.32–1.25; *P* = 0.184), 0.99 for asthma and hay fever (95% CI 0.72–1.36, *P* = 0.951) and 0.92 for self-reported allergy (95% CI 0.69–1.22; *P* = 0.540), with intercepts of −0.004 (95% CI −0.014 to 0.006, *P* = 0.396), 0.027 (95% CI 0.001 to 0.053, *P* = 0.042), −0.007 (95% CI −0.030 to 0.016, *P* = 0.542) and 0.017 (95% CI 0.003–0.031, *P* = 0.018). Collectively, these findings provide possible evidence of systematic bias in the IVW estimate for IgE level and self-reported allergy, which might have arisen through overall unbalanced horizontal pleiotropy. There was no such evidence for such pleiotropy in respect of atopic dermatitis.

We explored the possibility that a relationship between atopy and glioma might be subtype specific, considering GBM and non-GBM separately. Imposing a stronger significance threshold of *P* = 0.00625 (0.05/8, to correct for testing four traits over two outcomes), no histology-specific associations were shown by the IVW method between asthma and hay fever, IgE levels and self-reported allergy and glioma risk, with the respective ORs for the IVW method being 0.97, 0.92 and 1.04 for GBM tumours, and 0.96, 0.97 and 1.04 for non-GBM tumours (Additional file 7: Table S6). For atopic dermatitis, a significant OR of 0.94 (95% CI 0.90–0.98, *P* = 0.004) was shown for GBM but not for non-GBM (OR 0.98, 95% CI 0.93–1.03, *P* = 0.421). The association between atopic dermatitis and risk of GBM was also apparent in the MLE analysis, which provided an OR of 0.94 (95% CI 0.91–0.97, *P* = 2.17 × 10^− 4^). MR-Egger regression provided for an intercept of −0.007 (95% CI −0.019 to 0.005, *P* = 0.247). As with the analysis of all glioma, the association between atopic dermatitis and GBM was weaker under the WME (OR 0.96, 95% CI 0.91–1.02, *P* = 0.172) and MBE (OR 0.95, 95% CI 0.90–1.01, *P* = 0.096) frameworks.

Although previously implemented in other studies [32, 38], ratio estimators may not fully recapitulate an estimate of the causal OR in the case of binary exposures, such as atopic dermatitis, and binary outcomes such as glioma [39]. We therefore evaluated, through simulation, whether the IVW, MLE, WME, MBE and MR-Egger methods provide reliable estimates of causal ORs. When no causal relationship between exposure and outcome was simulated, each MR method provided accurate estimates of the null relationship (Additional file 5: Table S5). Conversely, when a causal relationship was simulated, the magnitudes of the relationship estimates were weakly inflated in some instances (Additional file 5: Table S5), indicating the importance of considering additional evidence when evaluating causal relationships between binary exposures and binary outcomes.

## Discussion

To our knowledge, this is the first MR study evaluating a range of atopy-related traits with glioma risk. Overall, our results provide evidence for a causal protective effect of atopic dermatitis with GBM tumours, but do not provide evidence that asthma and hay fever, raised IgE levels, or self-reported allergy is protective against the risk of developing glioma.

Possible mechanisms explaining an observed inverse relation between the risk of atopic dermatitis and the risk of glioma have been suggested in previous papers [12], postulated to be the consequence of immune system hyperactivity. The question thus arises as to how such divergent findings for other atopic traits can be explained or reconciled, when they have been previously reported in high numbers.

A key assumption in MR is that the instrument affects glioma risk through its effect on a specific phenotype/exposure (*i.e.* atopic traits), and does not have a direct effect on glioma risk. We tested this assumption using MR-Egger regression and HEIDI-outlier analysis and found possible evidence of violation of this assumption for IgE and self-reported allergy. It is notable that self-reported allergy does not show an approximately quadratic response to correlation, in contrast to asthma and hay fever, atopic dermatitis and IgE level. This is likely to be a consequence of imprecise estimates of the association between SNPs and allergy, illustrating the inherent issue in attempting to make use of self-reported allergy data as an atopy-related trait.

The meta-analyses of published epidemiological observational studies has indeed provided strong evidence for an inverse relationship between atopy and glioma risk [40]. However, most of the support for such a relationship came from case-control studies [4]. A common limitation in retrospective studies of glioma has been the use of proxy respondents for patients with cognitive impairment, who may not remember past exposures accurately due to cognitive deficits [4]. Such issues are compounded by the fact that, across studies, multiple atopic traits have been assessed. The strength of support for a relationship seen across case-control studies contrasts markedly with the limited evidence for a relationship from prospective cohort-based analyses [7].

By inference, a relationship between long-term antihistamine use could theoretically provide supporting evidence, albeit indirect, that atopic-mediated mechanisms influence glioma risk. However, the impact of antihistamine use is difficult to disentangle from that of allergies, as these factors are highly correlated and few individuals without allergies use antihistamines regularly. Paradoxically, an increased risk for glioma associated with antihistamines, particularly among individuals with allergic conditions, has been found in some studies [41, 42].

Raised IgE levels and self-reported allergy suffer limitations as traits used to assess the effect of atopy on glioma risk as they are both variable over short time scales in their level of expression (in contrast to clinical diagnosis of atopic dermatitis). Further, allergies may develop later in life, and patients may not necessarily exhibit symptoms. This introduces the possibility of bias and error due to the time varying association of SNPs with the exposure. However, it has been suggested that seasonality does not have a significant effect [11].

An additional possible explanation for the lack of causal association between IgE levels and glioma risk seen in this study is that the causality is in fact reversed, which could result in epidemiological observational studies reporting inverse relationships [8, 9], but would not affect an MR analysis. Immunosuppression caused by glioblastoma is well documented [43, 44] and may lead to reduced expression of atopy. Furthermore, in addition to steroids, temozolomide therapy, routinely used to treat GBM nowadays, leads to reduced blood IgE levels [11].

Using data from large genetic consortia for multiple atopy-related traits and glioma risk has enabled us to more precisely test our study hypotheses than if we had used individual-level data from a smaller study. Through simulation scenarios, the IVW, MLE, WME, MBE and MR-Egger methods have been demonstrated to accurately estimate causal effects using summary-level data [28, 30, 31, 45]. However, using summary-level data instead of individual-level data limits the approaches that can be used to test the validity of genetic variants as IVs, as adjusting for measured covariates and assessing gene-environment interactions is generally not possible using summary-level data [46]. The first-stage F statistic was large (> 25 for all traits), and therefore weak instrument bias is unlikely.

Epidemiological observational studies have reported inverse relationships between atopy-related traits and glioma risk, with ORs in the range 0.43–0.96 for asthma [6, 47], 0.42–0.90 for atopic dermatitis [6, 47], 0.37–0.73 for IgE levels [8–10] and 0.47–0.69 for self-reported allergies [4, 5, 8]. Odds ratios for binary exposures estimated in this MR study represent the OR for the outcome disease per doubling in odds of the exposure disease, and the magnitudes of these causal effect estimates are therefore not directly comparable to those reported in observational studies.

Our MR analysis has several strengths. Firstly, by utilising the random allocation of genetic variants, we were able to overcome potential confounding and reverse causation that may bias estimates from observational studies. Secondly, given that a poor outcome from glioma is almost universal, it is unlikely that survival bias will have influenced study findings. Lastly, the findings from this study represent the association of a lifelong atopy with glioma in the general European population.

Nevertheless, our study does have limitations. Firstly, while it is entirely appropriate to implement different MR methods to assess the robustness of findings, they have a differing power to demonstrate associations, with the WME, MBE and MR-Egger methods having less power than IVW and MLE. Irrespective of such factors, our study only had 80% power to detect ORs of 1.16, 1.09, 1.16 and 1.22 for asthma and hay fever, atopic dermatitis, IgE level and self-reported allergy, respectively (Additional file 4: Table S4), due to the very low proportion of variability in the atopy-related traits explained by the SNPs used. Hence, we cannot exclude the possibility that these traits influence glioma risk, albeit modestly. To explore this possibility, will require additional IVs and larger sample sizes affording increased power. Furthermore, it is possible that an effect of atopy on glioma risk might be mediated through mechanisms associated with a trait that we have not captured by using MR to assess asthma and hay fever and self-reported allergy. Secondly, a weakness of the two-sample MR strategy is that it does not allow examination of non-linear relationships between exposures and outcomes. Finally, we have sought to examine whether bias could be introduced when considering a binary exposure for a binary outcome. Although in our simulation study we found no evidence of bias when estimating non-causal relationships, we did not extend our analysis to consider the potential impact of invalid SNPs.

## Conclusions

In conclusion, our investigation does not provide strong evidence for a relationship between atopy-related diseases and risk of developing glioma, but findings do not preclude a small effect for atopic dermatitis. Our analysis also serves to illustrate the value of using several MR methods to derive robust conclusions.

## Additional files

Additional file 1: **Figure S1.** Forest plot of Wald odds ratios (ORs) and 95% confidence intervals generated from single nucleotide polymorphisms (SNPs) associated with atopic dermatitis, including rs909341. ORs for individual SNPs are listed according to magnitude of effect in the instrumental variable analysis and are presented with pooled effects using the inverse-variance weighting method. Squares represent the point estimate, and the bars are the 95% confidence intervals. (DOCX 89 kb)

Additional file 2: **Table S1.** Summary of the eight glioma genome-wide association studies. (XLSX 29 kb)

Additional file 3: **Table S2.** Table of single nucleotide polymorphisms (SNPs) reported in the NHGRI-EBI Genome-wide Association Studies Catalog for each trait, with correlations between SNPs. (XLSX 48 kb)

Additional file 4: **Table S3.** Percentage of variance explained by the combined sets of single nucleotide polymorphisms used as instrumental variables. (XLSX 33 kb)

Additional file 5: **Table S4.** Range of odds ratios for which study had < 80% power, for each atopy-related trait (*P* = 0.05, two-sided). (XLSX 9 kb)

Additional file 6: **Table S5.** Simulation analyses. (XLSX 28 kb)

Additional file 7: **Table S6.** Inverse-variance weighting, maximum likelihood estimation, weighted median estimate, mode-based estimate and Mendelian randomisation-Egger test results for combined atopy-related instrumental variables and glioma subtypes. (XLSX 39 kb)

## Abbreviations

CI: confidence interval
GBM: glioblastoma
GWAS: genome-wide association study
IV: instrumental variable
IVW: inverse-variance weighting
MBE: mode-based estimate
MLE: maximum likelihood estimation
MR: Mendelian randomisation
OR: odds ratio
SNP: single nucleotide polymorphism
WHO: World Health Organization
WME: weighted median estimate

## References

[1] Ostrom QT, Gittleman H, Xu J, Kromer C, Wolinsky Y, Kruchko C, Barnholtz-Sloan JS (2016). CBTRUS Statistical Report: primary brain and other central nervous system tumors diagnosed in the United States in 2009-2013. Neuro Oncol.

[2] Ostrom QT, Bauchet L, Davis FG, Deltour I, Fisher JL, Langer CE, Pekmezci M, Schwartzbaum JA, Turner MC, Walsh KM (2014). The epidemiology of glioma in adults: a “state of the science” review. Neuro Oncol.

[3] Wiemels JL, Wiencke JK, Sison JD, Miike R, McMillan A, Wrensch M (2002). History of allergies among adults with glioma and controls. Int J Cancer.

[4] Johansen C, Schüz J, Andreasen A-MS, Dalton SO (2017). Study designs may influence results: the problems with questionnaire-based case–control studies on the epidemiology of glioma. Br J Cancer.

[5] Schlehofer B, Blettner M, Preston-Martin S, Niehoff D, Wahrendorf J, Arslan A, Ahlbom A, Choi WN, Giles GG, Howe GR (1999). Role of medical history in brain tumour development. Results from the international adult brain tumour study. Int J Cancer.

[6] Cicuttini FM, Hurley SF, Forbes A, Donnan GA, Salzberg M, Giles GG, McNeil JJ (1997). Association of adult glioma with medical conditions, family and reproductive history. Int J Cancer.

[7] Schwartzbaum J, Jonsson F, Ahlbom A, Preston-Martin S, Lonn S, Soderberg KC, Feychting M (2003). Cohort studies of association between self-reported allergic conditions, immune-related diagnoses and glioma and meningioma risk. Int J Cancer.

[8] Wiemels JL, Wiencke JK, Patoka J, Moghadassi M, Chew T, McMillan A, Miike R, Barger G, Wrensch M (2004). Reduced immunoglobulin E and allergy among adults with glioma compared with controls. Cancer Res.

[9] Schlehofer B, Siegmund B, Linseisen J, Schuz J, Rohrmann S, Becker S, Michaud D, Melin B, Bas Bueno-de-Mesquita H, Peeters PH (2011). Primary brain tumours and specific serum immunoglobulin E: a case-control study nested in the European Prospective Investigation into Cancer and Nutrition cohort. Allergy.

[10] Calboli FC, Cox DG, Buring JE, Gaziano JM, Ma J, Stampfer M, Willett WC, Tworoger SS, Hunter DJ, Camargo CA (2011). Prediagnostic plasma IgE levels and risk of adult glioma in four prospective cohort studies. J Natl Cancer Inst.

[11] Wiemels JL, Wilson D, Patel C, Patoka J, McCoy L, Rice T, Schwartzbaum J, Heimberger A, Sampson JH, Chang S (2009). IgE, allergy, and risk of glioma: update from the San Francisco Bay Area Adult Glioma Study in the temozolomide era. Int J Cancer.

[12] Linos E, Raine T, Alonso A, Michaud D (2007). Atopy and risk of brain tumors: a meta-analysis. J Natl Cancer Inst.

[13] Davey Smith G, Hemani G (2014). Mendelian randomization: genetic anchors for causal inference in epidemiological studies. Hum Mol Genet.

[14] Meyers DA, Xu J, Postma DS, Levitt RC, Bleecker ER (1995). Two locus segregation and linkage analysis for total serum IgE levels. Clin Exp Allergy.

[15] Wilkinson J, Grimley S, Collins A, Simon Thomas N, Holgate ST, Morton N (1998). Linkage of asthma to markers on chromosome 12 in a sample of 240 families using quantitative phenotype scores. Genomics.

[16] Portelli MA, Hodge E, Sayers I (2015). Genetic risk factors for the development of allergic disease identified by genome-wide association. Clin Exp Allergy.

[17] Pierce BL, Burgess S (2013). Efficient design for Mendelian randomization studies: subsample and 2-sample instrumental variable estimators. Am J Epidemiol.

[18] Melin BS, Barnholtz-Sloan JS, Wrensch MR, Johansen C, Il'yasova D, Kinnersley B, Ostrom QT, Labreche K, Chen Y, Armstrong G (2017). Genome-wide association study of glioma subtypes identifies specific differences in genetic susceptibility to glioblastoma and non-glioblastoma tumors. Nat Genet.

[19] Granada M, Wilk JB, Tuzova M, Strachan DP, Weidinger S, Albrecht E, Gieger C, Heinrich J, Himes BE, Hunninghake GM (2012). A genome wide association study of plasma total IgE concentration in the Framingham Heart Study. J Allergy Clin Immunol.

[20] Baurecht H, Hotze M, Brand S, Büning C, Cormican P, Corvin A, Ellinghaus D, Ellinghaus E, Esparza-Gordillo J, Fölster-Holst R (2015). Genome-wide comparative analysis of atopic dermatitis and psoriasis gives insight into opposing genetic mechanisms. Am J Hum Genet.

[21] Paternoster L, Standl M, Waage J, Baurecht H, Hotze M, Strachan DP, Curtin JA, Bønnelykke K, Tian C, Takahashi A (2015). Multi-ethnic genome-wide association study of 21,000 cases and 95,000 controls identifies new risk loci for atopic dermatitis. Nat Genet.

[22] Schaarschmidt H, Ellinghaus D, Rodríguez E, Kretschmer A, Baurecht H, Lipinski S, Meyer-Hoffert U, Harder J, Lieb W, Novak N (2015). A genome-wide association study reveals 2 new susceptibility loci for atopic dermatitis. J Allergy Clin Immunol.

[23] Weidinger S, Willis-Owen SAG, Kamatani Y, Baurecht H, Morar N, Liang L, Edser P, Street T, Rodriguez E, O'Regan GM (2013). A genome-wide association study of atopic dermatitis identifies loci with overlapping effects on asthma and psoriasis. Hum Mol Genet.

[24] Ferreira MA, Matheson MC, Tang CS, Granell R, Ang W, Hui J, Kiefer AK, Duffy DL, Baltic S, Danoy P (2014). Genome-wide association analysis identifies 11 risk variants associated with the asthma with hay fever phenotype. J Allergy Clin Immunol.

[25] Ramasamy A, Curjuric I, Coin LJ, Kumar A, McArdle WL, Imboden M, Leynaert B, Kogevinas M, Schmid-Grendelmeier P, Pekkanen J (2011). A genome-wide meta-analysis of genetic variants associated with allergic rhinitis and grass sensitization and their interaction with birth order. J Allergy Clin Immunol.

[26] Hinds DA, McMahon G, Kiefer AK, Do CB, Eriksson N, Evans DM, St Pourcain B, Ring SM, Mountain JL, Francke U (2013). A genome-wide association meta-analysis of self-reported allergy identifies shared and allergy-specific susceptibility loci. Nat Genet.

[27] Louis DN, Perry A, Reifenberger G, von Deimling A, Figarella-Branger D, Cavenee WK, Ohgaki H, Wiestler OD, Kleihues P, Ellison DW (2016). The 2016 World Health Organization Classification of Tumors of the Central Nervous System: a summary. Acta Neuropathol.

[28] Burgess S, Butterworth A, Thompson SG (2013). Mendelian randomization analysis with multiple genetic variants using summarized data. Genet Epidemiol.

[29] Zhu Z, Zheng Z, Zhang F, Wu Y, Trzaskowski M, Maier R, Robinson M, McGrath J, Visscher P, Wray N (2018). Causal associations between risk factors and common diseases inferred from GWAS summary data. Nat Commun.

[30] Bowden J, Davey Smith G, Haycock PC, Burgess S (2016). Consistent estimation in Mendelian randomization with some invalid instruments using a weighted median estimator. Genet Epidemiol.

[31] Hartwig FP, Smith GD, Bowden J (2017). Robust inference in summary data Mendelian randomization via the zero model pleiotropy assumption. Int J Epidemiol.

[32] Gage SH, Jones HJ, Burgess S, Bowden J, Davey Smith G, Zammit S, Munafo MR (2017). Assessing causality in associations between cannabis use and schizophrenia risk: a two-sample Mendelian randomization study. Psychol Med.

[33] Burgess S (2014). Sample size and power calculations in Mendelian randomization with a single instrumental variable and a binary outcome. Int J Epidemiol.

[34] Paternoster L, Standl M, Johannes W, Baurecht H, Hotze M, Strachan DP, Curtin JA (2015). Multi-ancestry genome-wide association study of 21,000 cases and 95,000 controls identifies new risk loci for atopic dermatitis. Nat Genet.

[35] Weidinger S, Gieger C, Rodriguez E, Baurecht H, Mempel M, Klopp N, Gohlke H, Wagenpfeil S, Ollert M, Ring J (2008). Genome-wide scan on total serum IgE levels identifies FCER1A as novel susceptibility locus. PLoS Genet.

[36] Ramasamy A, Kuokkanen M, Vedantam S, Gajdos ZK, Couto Alves A, Lyon HN, Ferreira MAR, Strachan DP, Zhao JH, Abramson MJ (2012). Genome-wide association studies of asthma in population-based cohorts confirm known and suggested loci and identify an additional association near HLA. PLoS One.

[37] MacArthur J, Bowler E, Cerezo M, Gil L, Hall P, Hastings E, Junkins H, McMahon A, Milano A, Morales J (2017). The new NHGRI-EBI Catalog of published genome-wide association studies (GWAS Catalog). Nucleic Acids Res.

[38] Ahmad OS, Morris JA, Mujammami M, Forgetta V, Leong A, Li R, Turgeon M, Greenwood CM, Thanassoulis G, Meigs JB (2015). A Mendelian randomization study of the effect of type-2 diabetes on coronary heart disease. Nat Commun.

[39] Palmer TM, Sterne JA, Harbord RM, Lawlor DA, Sheehan NA, Meng S, Granell R, Smith GD, Didelez V (2011). Instrumental variable estimation of causal risk ratios and causal odds ratios in Mendelian randomization analyses. Am J Epidemiol.

[40] Amirian ES, Zhou R, Wrensch MR, Olson SH, Scheurer ME, Il’yasova D, Lachance D, Armstrong GN, McCoy LS (2016). Approaching a scientific consensus on the association between allergies and glioma risk: a report from the Glioma International Case-Control Study. Cancer Epidemiol Biomarkers Prev.

[41] Scheurer ME, El-Zein R, Thompson PA, Aldape KD, Levin VA, Gilbert MR, Weinberg JS, Bondy ML (2008). Long-term anti-inflammatory and antihistamine medication use and adult glioma risk. Cancer Epidemiol Biomarkers Prev.

[42] Amirian ES, Marquez-Do D, Bondy ML, Scheurer ME (2013). Antihistamine use and immunoglobulin E levels in glioma risk and prognosis. Cancer Epidemiol.

[43] Razavi S-M, Lee KE, Jin BE, Aujla PS, Gholamin S, Li G (2016). Immune evasion strategies of glioblastoma. Front Surg.

[44] Gustafson MP, Lin Y, New KC, Bulur PA, O'Neill BP, Gastineau DA, Dietz AB (2010). Systemic immune suppression in glioblastoma: the interplay between CD14(+)HLA-DR(lo/neg) monocytes, tumor factors, and dexamethasone. Neuro Oncol.

[45] Bowden J, Davey Smith G, Burgess S (2015). Mendelian randomization with invalid instruments: effect estimation and bias detection through Egger regression. Int J Epidemiol.

[46] Burgess S, Bowden J, Fall T, Ingelsson E, Thompson SG (2017). Sensitivity analyses for robust causal inference from Mendelian randomization analyses with multiple genetic variants. Epidemiology.

[47] Il'yasova D, McCarthy B, Marcello J, Schildkraut JM, Moorman PG, Krishnamachari B, Ali-Osman F, Bigner DD, Davis F (2009). Association between glioma and history of allergies, asthma, and eczema: a case-control study with three groups of controls. Cancer Epidemiol Biomarkers Prev.

