# Supplementary material for: Impact of atopy on risk of glioma: a Mendelian randomisation study
Source: BMC Med. 2018 Mar 15;16:42. doi: 10.1186/s12916-018-1027-5 (PMC5853158; doi:10.1186/s12916-018-1027-5)
Supplement: Supplementary file 1 — Figure S1. Forest plot of Wald odds ratios (ORs) and 95% confidence intervals generated from single nucleotide polymorphisms (SNPs) associated with atopic dermatitis, including rs909341. ORs for individual SNPs are listed according to magnitude of effect in the instrumental variable analysis and are presented with pooled effects using the inverse-variance weighting method. Squares represent the point estimate, and the bars are the 95% confidence intervals. (DOCX 89 kb) [file 12916_2018_1027_MOESM1_ESM.docx]

**Figure S1: Forest plot of Wald ORs and 95% confidence intervals generated from SNPs associated with atopic dermatitis, including rs909341.** ORs for individual SNPs are listed according to magnitude of effect in the instrumental variable analysis and are presented with pooled effects using the IVW method. Squares represent the point estimate, and the bars are the 95% confidence intervals.
